# Case report: Mutations in *DNAJC30* causing autosomal recessive Leber hereditary optic neuropathy are common amongst Eastern European individuals

**DOI:** 10.3389/fneur.2023.1292320

**Published:** 2023-12-01

**Authors:** Toby Charles Major, Eszter Sara Arany, Katherine Schon, Magdolna Simo, Veronika Karcagi, Jelle van den Ameele, Patrick Yu Wai Man, Patrick F. Chinnery, Catarina Olimpio, Rita Horvath

**Affiliations:** ^1^School of Clinical Medicine, University of Cambridge, Cambridge, United Kingdom; ^2^Department of Clinical Neurosciences, School of Clinical Medicine, University of Cambridge, Cambridge, United Kingdom; ^3^Department of Clinical Genetics, East Anglian Medical Genetics Service, Addenbrooke's Hospital, Cambridge, United Kingdom; ^4^University Clinic of Neurology, Semmelweis University, Budapest, Hungary; ^5^Istenhegyi Genetic Diagnostic Center, Budapest, Hungary; ^6^NIHR Biomedical Research Centre, Moorfields Eye Hospital & UCL Institute of Ophthalmology, London, United Kingdom; ^7^Cambridge Eye Unit, Addenbrooke's Hospital, Cambridge University Hospitals, Cambridge, United Kingdom

**Keywords:** Leber hereditary optic neuropathy (LHON), mitochondrial LHON (mtLHON), autosomal recessive LHON (arLHON), DNA-J heat shock protein family (Hsp40) member C30 (DNAJC30), c.152A>G (p.Tyr51Cys), recessive optic neuropathy, idebenone

## Abstract

**Background:**

Leber Hereditary Optic Neuropathy (LHON) is the most common inherited mitochondrial disease characterized by bilateral, painless, subacute visual loss with a peak age of onset in the second to third decade. Historically, LHON was thought to be exclusively maternally inherited due to mutations in mitochondrial DNA (mtDNA); however, recent studies have identified an autosomal recessive form of LHON (arLHON) caused by point mutations in the nuclear gene, *DNAJC30*.

**Case Presentations:**

In this study, we report the cases of three Eastern European individuals presenting with bilateral painless visual loss, one of whom was also exhibiting motor symptoms. After a several-year-long diagnostic journey, all three patients were found to carry the homozygous c.152A>G (p.Tyr51Cys) mutation in *DNAJC30*. This has been identified as the most common arLHON pathogenic variant and has been shown to exhibit a significant founder effect amongst Eastern European individuals.

**Conclusion:**

This finding adds to the growing cohort of patients with arLHON and demonstrates the importance of *DNAJC30* screening in patients with molecularly undiagnosed LHON, particularly in Eastern European individuals. It is of heightened translational significance as patients diagnosed with arLHON exhibit a better prognosis and response to therapeutic treatment with the co-enzyme Q10 analog idebenone.

## Introduction

### Leber hereditary optic neuropathy

Leber Hereditary Optic Neuropathy (LHON) is the most common disease caused by mutations in mitochondrial DNA (mtDNA) and typically manifests in the second or third decade of life (1). LHON is characterized by acute or subacute bilateral painless visual loss, often accompanied by dyschromatopsia and central or centrocecal scotomas (1, 2). The penetrance of LHON does not appear to be related to the mitochondrial mutation load; however, the incidence of LHON manifestation is 3–5 times higher in male individuals (3, 4), which may be attributable to lifestyle and hormonal factors (3–6).

### Mitochondrial LHON

The majority of LHON cases, approximately 90–95%, are caused by mutations in mtDNA that are maternally inherited. This mitochondrial form of LHON (mtLHON) is most commonly associated with one of three mutations, in order of frequency: m.11778G>A (p.Arg340His) in MT-ND4, m.14484T>C (p.Met64Val) in MT-ND6, and m.3460G>A (p.Ala52Thr) in MT-ND1 (1, 7). In addition to these, 30 other rare variants in mtDNA are known to be associated with the disease (8).

mtLHON is thought to occur due to dysfunction in complex I (CI) of the mitochondrial electron transport chain, which leads to decreased adenosine triphosphate (ATP) synthesis and the increased production of reactive oxygen species (ROS). Damage to cellular components caused by ROS generation as well as the increased energy demands of the retinal ganglion cells (RGCs) renders them particularly vulnerable to declining levels of ATP, which is then thought to culminate in cellular death and axonal degeneration (1, 7).

The prognosis of mtLHON can be improved by treatment with the coenzyme Q10 analog idebenone, which has recently been approved for use in patients with LHON in the United Kingdom (9). Idebenone facilitates the bypass of dysfunctional CI, thereby restoring ATP synthesis and increasing energy availability to the RGCs, thus improving visual symptoms (10–13). For reasons not yet understood, spontaneous visual recovery can also occur in some patients (14, 15).

### Autosomal recessive LHON

More recently, a growing cohort of patients have been identified with an autosomal recessive form of LHON (arLHON). These patients present with similar visual symptoms as are seen in mtLHON (16) and show the same male predominance pattern (8.5:1 male:female ratio) (17). The majority of arLHON cases can be attributed to point mutations in the nuclear gene *DNAJC30* (16), but a number of other nuclear genes, including *NDUFS2* (18), *NDUFA12* (19), *MCAT* (20), and MECR (21), have also been implicated.

To date, there have been six reported pathogenic variants in *DNAJC30* associated with arLHON. These include three missense variants c.152A>G (p.Tyr51Cys), c.232C>T (p.Pro78Ser) (16), and c.302T>A (p.Leu101Gln) (16), a nonsense variant c.610G>T (p.Glu204^*^), a 3 bp in-frame deletion c.230_232del (p.His77del), and a frameshift variant c.130_131del (p.Ser44ValfsTer8) (22). Accounting for ≥90% of these cases is the c.152A>G (p.Tyr51Cys) point mutation. This specific mutation has been shown to exhibit a strong founder effect amongst individuals of Eastern European descent (16, 23) and is thought to have arisen from a common ancestor around 85 generations ago. In addition to this, more recent data have suggested that this mutation is also more prevalent than predicted in other European populations, including Central Europeans (24) and Estonians (25).

Although arLHON-causing mutations are less common than the mitochondrial variants as a whole, the individual prevalence of the c.152A>G (p.Tyr51Cys) variant in *DNAJC30* is not far below that of the m.14484T>C (p.Met64Val) in the MT-ND6 mutation causing mtLHON (24). In addition, a retrospective study has suggested that variants in *DNAJC30* can account for up to 7.7% of clinically apparent LHON (24). Aside from arLHON, mutations in *DNAJC30* have also been found in patients with Leigh syndrome (26) and in one family who were exhibiting a movement disorder phenotype (22).

The largest cohort of investigated patients and published case studies suggest that arLHON may have subtle but distinct clinical features compared to mtLHON. arLHON is likely to present at a younger age (24, 27), with a shorter interval between the onset of symptoms in both eyes (24) compared to mtLHON. Furthermore, arLHON patients may have a more favorable prognosis (24, 27) and have been shown to exhibit a better response to idebenone (17, 28).

The *DNAJC30* gene encodes a chaperone protein mainly expressed in neurons, which has been shown to interact with multiple components of the electron transport chain. The protein is thought to be involved in the regulation of CI by promoting the exchange of CI subunits, which have been exposed to higher levels of oxidative damage in order to maintain a high-functioning state of CI (16). In addition, it has been shown to interact with the H^+^-loading component of complex V (29). Whilst there is still some discussion about the prevalent function of *DNAJC30*, the impaired assembly, maintenance, and turnover of CI are all thought to contribute to the observed arLHON phenotype (16) in patients with *DNAJC30* mutations.

Here, we present a case report of three patients from Eastern Europe who were found to have homozygous pathogenic variants in *DNAJC30*. All patients exhibited symptoms of LHON, and one of them also displayed a movement disorder phenotype.

## Case presentations

### Patient 1

A 35-year-old male Polish patient presented with bilateral painless visual loss that had developed over a 1-month period 2 years prior. He had also developed diplopia and gait disturbances. He was born in Poland to non-consanguineous parents before moving to the United Kingdom. There was no family history of similar symptoms, and he had one younger, healthy female sibling.

On examination, he was found to have reduced bilateral visual acuity, ophthalmoparesis, diplopia on the side gaze, and a right-sided nystagmus. He was also noted to exhibit mild sensorineural hearing loss. A peripheral neurological examination found mild gait ataxia and mild muscle weakness in the upper and lower limbs that were worse proximally. Deep tendon reflexes were present but mildly reduced with normal ankle jerks and down-going plantar reflexes. Tandem gait was difficult, and the patient exhibited a mildly positive Romberg sign. There was no evidence of dysarthria, dysmetria, or dysdiadochokinesia.

**Figure 1:**
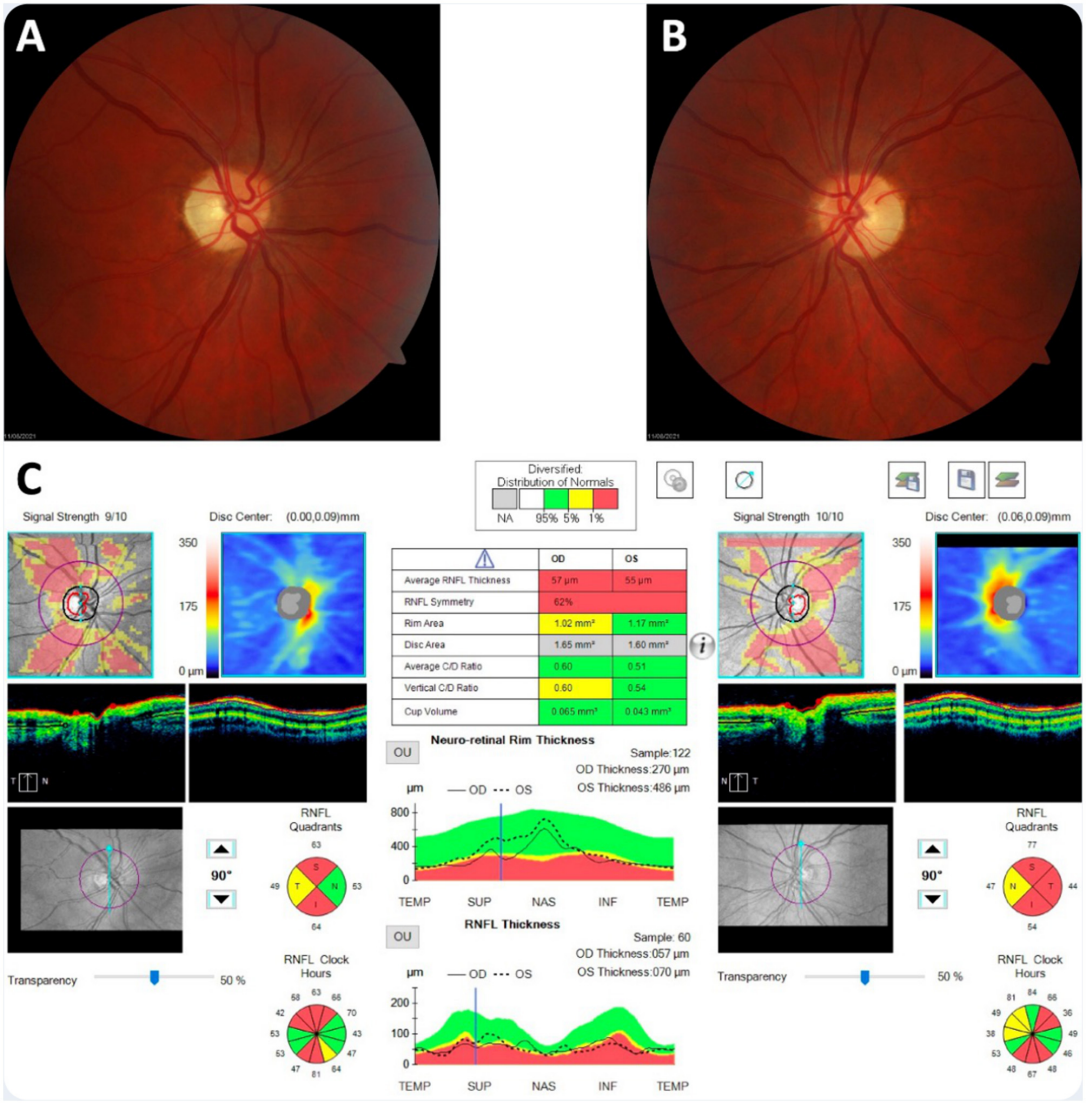
Neuro-ophthalmological findings 2 years after the onset of visual loss. **(A, B)** Bilateral optic atrophy was observed on dilated fundus examination. **(C)** Optical coherence tomography imaging showed significant thinning of the peripapillary retinal nerve fiber layer.

**Figure 2:**
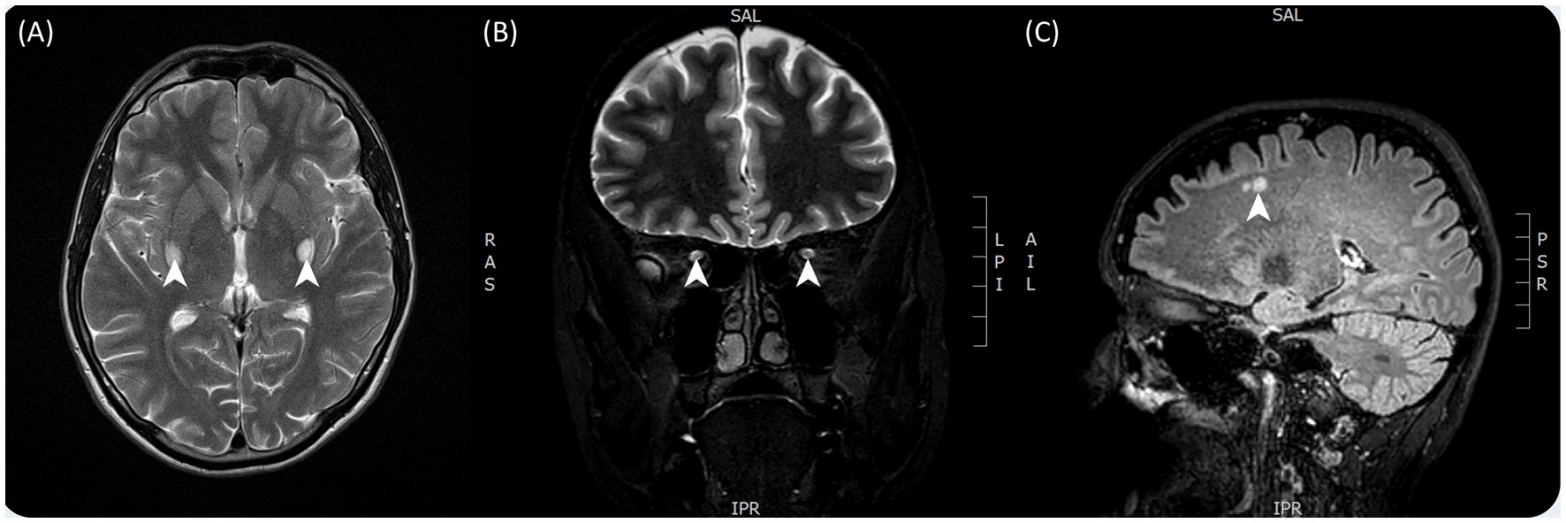
T2-weighted contrast-enhanced brain MRI images taken from both patients. **(A)** Taken from patient 1 and showing bilateral enhancements of the posterior basal ganglia in particular the putamen. **(B)** Taken from patient 2 and showing hyperintensity of the optic tracts bilaterally. **(C)** Taken from patient 2 and showing hyperintense lesions in the subcortical layer of the cerebrum.

Prior investigations had concluded that the patient had severe bilateral optic atrophy with an almost total absence of retinal ganglion cells in the macula of both eyes (Figure 1). A contrast-enhanced brain MRI revealed symmetrical enhancements of the posterior basal ganglia, which raised the suspicion of mitochondrial disease (Figure 2A).

Ischemic vascular, compressive, and acquired nutritional causes of bilateral visual loss were thoroughly investigated and ruled out based on brain imaging, blood work, and thorough history-taking and examination. The patient was negative for both aquaporin 4 (AQP-4) antibodies and myelin oligodendrocyte glycoprotein (MOG) antibodies. CSF analysis was unremarkable with normal lactate levels, and no demyelinating lesions were identified. The patient was also negative for anti-treponemal antibodies.

Carbon monoxide poisoning was also investigated, as this is a known cause of visual disturbance and increased T2-weighted MRI signal intensity in the posterior basal ganglia. However, this was ruled out after the patient's boiler was assessed by a gas service engineer, who found no carbon monoxide or other toxic fume production.

Hereditary causes of optic atrophy were also investigated. The patient was negative for the three most common mtLHON-causing mutations as well as for another 63 known hereditary optic atrophy-causing mutations across mitochondrial and autosomal genes, which were assessed using the NHS National Genomic Test Directory R41 Gene Panel. At the time, *DNAJC30* was not included in this panel. Given our patient's demographics, we decided to sequence a locus of his DNAJC30 gene known to contain the site of the c.152A>G (p.Tyr51Cys) mutation. Sequencing using the Sanger method revealed that the patient was homozygous for this pathogenic variant, confirming the diagnosis of arLHON.

At follow-up in the neurogenetics clinic, his symptoms had largely remained the same, with some minor improvement noted in his diplopia and tandem gait. Importantly, for this patient, the confirmation of his diagnosis allowed us to recommend treatment with idebenone. We were also able to offer genetic counseling to both the patient and his sister, as well as additional screening for her and her children to investigate further at-risk relatives.

Although idebenone (Raxone) was approved by the European Medicines Agency for the treatment of LHON, it has not been approved for reimbursement by NHS England (9). Therefore, it was not possible to commence the patient on idebenone, and he was unable to self-fund the treatment. At the last follow-up, the patient's visual acuity had improved spontaneously to 6/18 in his right eye and 6/60 in his left eye. His motor symptoms had not changed.

### Patient 2

A 49-year-old Hungarian woman presented to the clinic with bilateral painless visual loss that had begun 3 years prior. Her symptoms developed over a 3-month period, starting with involvement of the right eye before progressing to involve the left eye 2 months later. She had no other neurological symptoms, and there was no relevant family history.

On examination, she had bilaterally reduced visual acuity of 0.01 and 0.02 in the right and left eye, respectively. Her pupils were reactive, and there was no ophthalmoparesis or nystagmus. A full neurological examination found no additional neurological deficits.

Goldmann perimetry and visual evoked potentials testing concluded that the patient was suffering from severe bilateral optic atrophy with a large centrocecal scotoma extending upwards, temporally, and downwards beyond the blind spot and center in the right eye and a scotoma affecting the lower center and extending downward from it in the left eye.

Multiple contrast-enhanced MRIs indicated T2 signal enhancement with contrast accumulation in the right optic nerve and a similar but smaller signal enhancement on the left side, consistent with inflammation. On repeated imaging, contrast material accumulation resolved, but signal enhancement along the right optic nerve and tracts became more marked (Figure 2B). There were also some small, non-specific, hyper-enhancing lesions present in subcortical, intracerebral locations (multiple in the corona radiata and one in the cerebellum), reported as small ischemic lesions, probably not contributing to the patient's symptoms (Figure 2C). CSF protein levels were normal, as were the cell counts. CSF immunological tests were also normal, including AQP-4 and MOG antibodies. Treponema, HIV, Borrellia, HCV serology, and sACE levels were normal, and a thrombophilia panel was unremarkable.

Although no antibodies were identified in her case, inflammatory causes remained one of the top differential diagnoses, given the MRI findings and the initial clinical progression that were consistent with neuromyelitis optica (NMO). Initially, she was started on steroid treatment, which did not cause any change in her symptoms. She was commenced on a regular rituximab infusion, which did not cause any immediate improvement; however, she reported improved visual acuity after 2 years. The significant delay in symptom improvement after prolonged immunosuppressive therapy with steroids and rituximab made an inflammatory cause very unlikely.

This led to the consideration of hereditary causes of optic atrophy. The patient's mitochondrial DNA sample was negative for m.11778G>A (p.Arg340His) in MT-ND4, m.3460G>A (p.Ala52Thr) in MT-ND1, and m.14484T>C (p.Met64Val) in MT-ND6.

Prior to confirmation of the genetic diagnosis, this patient received genetic counseling, and her treatment was commenced by the clinical team in Hungary. Akin to the situation in the United Kingdom, there is currently no reimbursement offered by the national health insurance system in Hungary for idebenone therapy in LHON. She was however able to self-fund the treatment comprising 200 mg of coenzyme Q10 and 450 mg of idebenone. At the last follow-up appointment, the patient's disease was stable with no evidence of progression. Her visual acuity was slightly improved in the right eye (0.05) and significantly improved in the left eye (0.8). Sanger sequencing in our lab later confirmed that the patient is homozygous for the c.152A>G (p.Tyr51Cys) pathogenic variant.

### Patient 3

A 45-year-old Polish man, living in the United Kingdom, presented with sequential painless visual loss at the age of 31. His symptoms developed over a 4-month period, starting with involvement of the left eye, followed by involvement of the right eye 3 months later. He had a history of migraines but no other neurological symptoms. There was no relevant family history, and his parents were non-consanguineous. He smoked cigarettes from the age of 17 to 31 (1 packet a day) and drank beer at the weekend. He had a near-complete loss of central and color vision and was severely sight impaired. Routine investigation, including brain MRI, testing for common LHON mutations, POLG sequencing, and OPA1 sequencing, did not reveal a cause. A quadriceps muscle biopsy showed one COX-deficient fiber and no ragged red fibers. Whole mtDNA genome sequencing was normal. He was recruited for the 100,000 Genomes Project. The optic neuropathy panel applied did not include *DNAJC30*, so he did not initially receive a diagnosis through the Genomic Medicine Centre. However, his homozygous pathogenic variant was discovered by researchers in the research environment and fed back to his clinical team.

Examination at the age of 45 showed severe visual impairment, with him being able to see only hand motions. He had bilateral afferent pupillary defects and scored 0/15 bilaterally on the Ishihara testing. He had pale optic disks bilaterally. Repeated optical coherence tomography examinations showed stable optic atrophy. He was taking Coenzyme Q10 400 mg BD. He was aware of idebenone but was unable to self-fund the treatment.

## Materials and methods

### *DNAJC30* sequencing

For Patients 1 and 2, DNA was extracted from stored blood samples. The locus known to contain the site of the c.152A>G (p.Tyr51Cys) mutation was amplified using the polymerase chain reaction. The sequences of the forward and reverse primers used for amplification were 5′-GCTGTTACCTTGGAGGTTGC-3′ and 5′-AAGTTGAACATCGTGCGGTTG-3′, respectively. Sanger sequencing was then used to sequence the amplified fragment and interrogate the sequence for the presence of the c.152A>G mutation.

### 100,000 genomes project

Patient 3 was identified based on data collected as part of the 100,000 Genomes Project. This project recruited participants with genetically undiagnosed rare diseases between 2015 and 2018 through Genomic Medicine Centres in the United Kingdom (30). Patient 3 was recruited under the category of inherited optic neuropathies. DNA extraction, sequencing, alignment, and variant calling were performed as previously described (31, 32). A virtual gene panel for inherited optic neuropathies was applied (33). Variants were classified into four “tier” groups according to the probability of being causative (31, 32). We analyzed tier 1–3 variants in the *DNAJC30* gene and identified that Patient 3 had a homozygous pathogenic variant. We contacted his clinician about the diagnosis and offered to see the patient in the context of mitochondrial clinical research studies.

## Results

**Figure 3:**
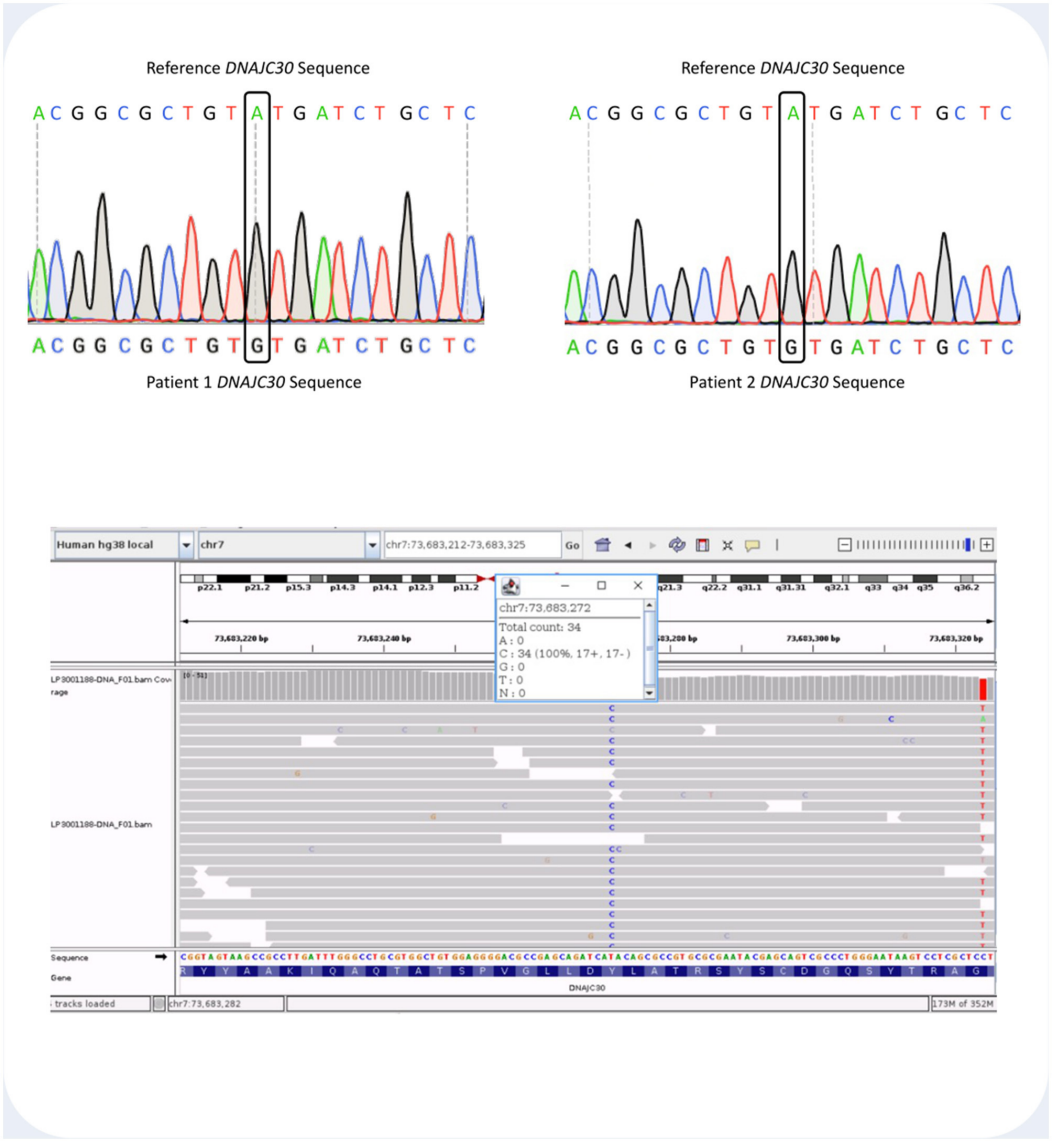
**(Top)** Sanger sequencing histogram readout showing the DNAJC30 loci containing the site of the c.152A>G (p.Tyr51Cys) mutation in Patient 1 and 2. Above each histogram there is the DNAJC30 reference sequence. **(Bottom)** Integrative Genome Viewer (IGV) screenshot showing the aligned sequencing reads and the count details for Patient 3. The homozygous T>C variant at genomic coordinates GRCh38 chr7-73,683,272 represents the c.152A>G DNAJC30 pathogenic variant.

All three patients were identified as homozygotes for the c.152A>G mutation in *DNAJC30*. The region of the *DNAJC30* gene harboring the mutation is shown in Figure 3.

## Discussion

In this study, we report the cases of three Eastern European individuals with arLHON associated with the c.152A>G (p.Tyr51Cys) pathogenic variant in *DNAJC30*. This adds to the growing cohort of patients with arLHON and provides further evidence for the hypothesis of a significant founder effect associated with this variant. Interestingly, we find additional clinical evidence of motor involvement associated with the disease, with one of our patients exhibiting gait ataxia and proximal muscle weakness characteristic of Leigh syndrome. This may in future form part of the spectrum of clinical features that distinguish arLHON from mtLHON, alongside its apparent earlier age of onset and greater therapeutic response to idebenone. The main clinical symptom exhibited by Patient 1 was optic neuropathy; however, neurological examination detected some other subtle signs. The recent literature on potential genetic modifiers in nuclear-encoded complex I-related genes in patients with more severe and complex LHON raises the possibility of such an additional modifier in this patient. We could not detect any additional variant on the optic neuropathy panel containing NDUFA12, but we cannot exclude variants in other nuclear complex I genes (23). Identification of the DNAJC30 pathogenic variant in further patients may elucidate if these symptoms form part of the spectrum of clinical features in arLHON.

Recognizing arLHON remains a challenge, as the male predominance and low penetrance of the disease reduce the likelihood of symptom manifestation. This could mean that families harboring arLHON-associated *DNAJC30* mutations may go undetected for several generations. In addition, with the differential diagnosis of optic atrophy remaining wide, there are several different etiologies to consider, such as neuromyelitis optica, carbon monoxide poisoning, and infectious or vascular causes. These cases emphasize the importance of considering arLHON in patients with features of LHON but for whom genetic testing is not possible or has so far proved inconclusive. This is of heightened significance in individuals of Eastern European descent presenting with subacute, bilateral painless visual loss.

Similarly, the cases reported here strengthen the argument to include *DNAJC30* testing as part of the diagnostic work-up in patients presenting with bilateral optic atrophy/neuropathy. Until recently (March 2023), the NHS National Genomic Test Directory did not include testing of *DNAJC30* as part of its R41 (Optic Neuropathy) Gene Panel. Furthermore, despite this recent progress in testing, it still appears difficult for patients in the United Kingdom with arLHON to access idebenone treatment on the NHS as funding is not currently in place for the drug. Given the good responsiveness of arLHON to idebenone therapy compared with mtLHON, combined with the potential for timely intervention to prevent significant visual loss, action should be taken to make this treatment available. Similarly, *DNAJC30* genetic testing should be adopted wherever possible, especially in Europe and the United States, which have a large population of patients of Eastern European descent.

Further research may clarify the significance of the other nuclear genes (*NDUFS2, NDUFA12, MCAT*, and *MECR*) involved in CI assembly, stability, and fatty acid biosynthesis in the pathogenesis and prognosis of painless visual loss.

## Data availability statement

The datasets presented in this article are not readily available because of ethical and privacy restrictions. Requests to access the datasets should be directed to the corresponding author.

## Ethics statement

Ethical review and approval was not required for the study on human participants in accordance with the local legislation and institutional requirements. The human samples used in this study were acquired from a by- product of routine care or industry. All patients gave written informed consent for the publication of this study.

## Author contributions

TM: Data curation, Formal analysis, Investigation, Methodology, Writing—original draft, Writing—review & editing. EA: Investigation, Writing—original draft, Writing—review & editing. KS: Data curation, Investigation, Methodology, Writing—review & editing. MS: Data curation, Writing—review & editing. VK: Data curation, Writing—review & editing. JA: Writing—review & editing. PY: Data curation, Resources, Writing—review & editing. PC: Supervision, Writing - review & editing. CO: Writing—review & editing. RH: Conceptualization, Data curation, Funding acquisition, Investigation, Methodology, Resources, Supervision, Writing—review & editing.
